# Multiscale Analysis of Composite Structures with Artificial Neural Network Support for Micromodel Stress Determination

**DOI:** 10.3390/ma17010154

**Published:** 2023-12-27

**Authors:** Wacław Kuś, Waldemar Mucha, Iyasu Tafese Jiregna

**Affiliations:** Department of Computational Mechanics and Engineering, Silesian University of Technology, 44-100 Gliwice, Poland; waclaw.kus@polsl.pl (W.K.); iyasu.tafese.jiregna@polsl.pl (I.T.J.)

**Keywords:** multiscale modeling, finite element method, homogenization, artificial neural network, composite material, fiber-reinforced composite, machine learning

## Abstract

Structures made of heterogeneous materials, such as composites, often require a multiscale approach when their behavior is simulated using the finite element method. By solving the boundary value problem of the macroscale model, for previously homogenized material properties, the resulting stress maps can be obtained. However, such stress results do not describe the actual behavior of the material and are often significantly different from the actual stresses in the heterogeneous microstructure. Finding high-accuracy stress results for such materials leads to time-consuming analyses in both scales. This paper focuses on the application of machine learning to multiscale analysis of structures made of composite materials, to substantially decrease the time of computations of such localization problems. The presented methodology was validated by a numerical example where a structure made of resin epoxy with randomly distributed short glass fibers was analyzed using a computational multiscale approach. Carefully prepared training data allowed artificial neural networks to learn relationships between two scales and significantly increased the efficiency of the multiscale approach.

## 1. Introduction

Multiscale analysis has been a major research topic for decades, focusing on the role of material microstructures in macroscale mechanical behaviors [1]. Modern heterogeneous materials like fiber-reinforced [2] or multimetallic composites [3] are gaining more and more popularity in engineering structures due to their application-specific tailored properties. To accurately describe their microstructural behavior, numerical methods such as the multiscale finite element method [4,5,6,7], asymptotic homogenization method [8,9,10], coarse-graining technique [11], and finite element and Fast Fourier Transforms method [12] have been developed. High-fidelity microstructure material modeling is essential for macroscale structural analysis prediction. However, this raises a computational efficiency issue. In the classical multiscale finite element method, each integration point queries mechanistic information from an evolving microstructure using a high-fidelity representative volume element (RVE). This results in intractable computational costs, especially for industrial applications. The trade-off between accuracy and efficiency in classical multiscale simulation has been reported by many researchers [13,14,15,16]. Despite its potential, computational cost is a fundamental issue in large-scale structural analysis applications.

Furthermore, machine learning techniques have shown great potential in accelerating multiscale simulations by learning microstructural responses through data from numerical simulations. Machine learning methods, like Artificial Neural Networks (ANNs), have been successfully used for speeding up homogenization methods, that is, estimating the collective behavior of a heterogenous material and extracting effective material properties of equivalent homogenous material at the macroscopic scale. Le et al. [17] used ANNs for nonlinear homogenization. Lu et al. [18] utilized ANNs for the computational homogenization of the electric parameters of random graphene-polymer nanocomposites. In [19,20] recurrent neural networks were implemented for modeling the history-dependent nonlinear microlevel response. Mozaffar et al. [21] used deep learning to predict the plastic behavior of a given class of composite RVEs. Convolutional neural networks have also been successfully adapted to predict complex effective parameters for certain composite materials, like plastic [22] or anisotropic elastic [23]. Vlassis et al. [24] used graph neural networks to predict homogenized responses of polycrystals. In addition to these, the multiscale data-driven finite element-deep material network (FE-DMN) is a method that connects machine learning techniques with mechanics. This multiscale material modeling method correctly and efficiently predicts macroscale material responses compared to direct numerical simulation of the RVE [25]. DMN captures the complexity of microstructural interactions through a binary network structure with a two-layer mechanistic building block [26]. The FE-DMN architecture allows for the prediction of non-linear material behavior based on linear elastic training data [26]. Studies have explored potential applications of DMN, including 3D DMN architectures [27] for composite materials and metallic materials, a unified DMN database [28], extensions for multiphase composites [15], woven composites [29], porous microstructural materials [30], interfacial failure and damage analysis [31,32], and applications in multiscale simulation [13,14]. The data-driven FE-DMN method offers great opportunities for industrial applications [33].

A data-driven multiscale finite element method has been applied to composite materials and structures [16]. This method originates from data-driven computational mechanics [34], and is applied to a multiscale simulation framework. Similar data-driven multiscale methodologies have been conducted for biological and technological hierarchical materials [35,36] and granular materials [37]. Most importantly, a multiscale modeling technique based on a two-scale analysis approach was conducted [38], and multiscale bone modeling was presented. The important modeling process considered in the paper was the identification problem, formulated as an inverse problem that considered two-scale analysis.

The novelty of this study is that machine learning has been introduced in multiscale analysis to successfully speed up a new class of problem: localization. The research thesis is that a prediction model, based on ANNs, can significantly increase the efficiency of multi-scale modeling by substituting the computationally expensive FEM model of the microstructure. If the training data is carefully prepared, the ANN can be used to accurately estimate microstructural stresses, for a given composite material, at any point, for any macromodel geometry. The goal of this paper is to present a methodology for the application of the data-driven multiscale finite element method and artificial neural networks for the prediction of stress concentrations in composite structures. The values of stress concentrations in the microstructure of composite materials under load may be substantially higher than those obtained from structural macroscale analysis, where only homogenized material properties are considered. The microscale effects may be important when considering potential failure mechanisms in composite materials (microcracks, delamination, etc.). The presented approach of time-efficient determination of stresses in inclusions and matrix within composites could be used in the future, for example, in structural health monitoring (SHM) of modern structures [39,40], and especially in the variant of SHM known as Operational Load Monitoring processes (OLM, fatigue tracking) [41,42,43]. In OLM the amount and characteristics of load cycles that a structure endured in its operational environment are measured and registered. The point is to determine the remaining in-service life and maximize the usefulness of the structure before its replacement. Such processes are applied mainly in aerospace and civil engineering industries [44,45], and are often supported by machine learning [46,47,48,49]. As modern structures subjected to monitoring are often composite with a nonlinear relation between macroscale and microscale behavior, the authors suspect that efficient determination of microstructural behavior could improve the accuracy of SHM and OLM processes in the future.

To carry out the described goals, a computational multiscale approach with two-scale (microscale and macroscale) analysis of composite material was used. The representative volume element (RVE) was created for the microstructure with linear displacement boundary condition and the computational homogenization method was employed to obtain the averaged material properties of the microstructure [50,51]. Artificial neural networks (ANNs) were trained based on a dataset generated from a carefully prepared series of microscale analyses, to quickly and accurately determine the stresses in composite microstructure based on results obtained from macroscale finite element analysis. Substantial efficiency improvement was achieved.

The proposed methodology of substituting micromodel by ANN in multi-scale analysis is defined in Section 2. In Section 3 a numerical example is described where the previously defined methodology is verified. Conclusions and directions for future research are summarized in Section 4.

## 2. Materials and Methods

Multiscale modeling considers dependencies between scales and uses computational homogenization to replace heterogeneous materials with homogeneous ones [38]. This method is useful for periodic microstructures. The influence between scales is obtained by solving the boundary value problem in each scale using a numerical method [38,52].

**Figure 1:**
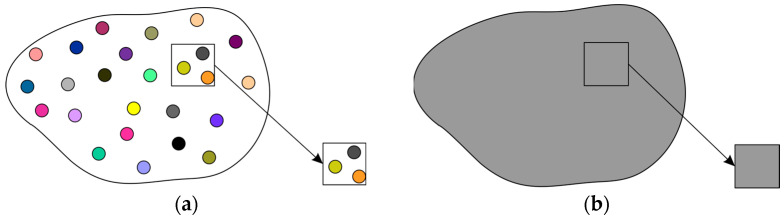
Macromodel with: (**a**) heterogeneous material and (**b**) homogeneous material.

**Figure 2:**
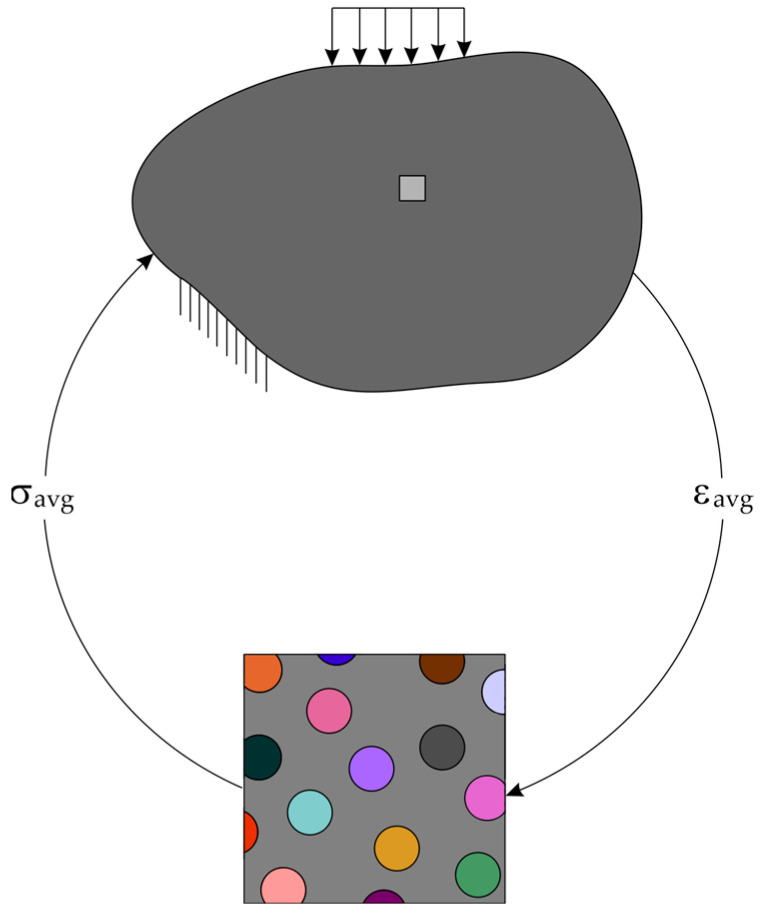
The two-scale computational homogenization for a composite material.

The heterogeneous material is replaced by the homogeneous material as shown in Figure 1. The heterogeneous periodic material can be modeled using a periodic microstructure model, namely the Representative Volume Element (*RVE*). The homogenization procedure allows one to obtain material properties for the macroscale based on results in the microscale. The two-scale computational homogenization method is illustrated in Figure 2. Macro strain values (average strain for microscale) are transferred to the micromodel for each integration point of finite elements. The microscale model—*RVE* is analyzed with boundary conditions defined by macrolevel average strains. The results of microscale analysis are used to compute average stresses which are transferred to the macroscale.

For *RVE*, the average strain and stress are given as follows:

(1) $$\varepsilon_{avg}=\frac{1}{\left|\Omega_{RVE}\right|}\underset{\Omega_{RVE}}{\int }\varepsilon d\Omega_{RVE}\;\sigma_{avg}=\frac{1}{\left|\Omega_{RVE}\right|}\underset{\Omega_{RVE}}{\int }\sigma d\Omega_{RVE}$$

where $\Omega_{RVE}$ is the domain of microscale model, $\varepsilon_{avg}$ is the average strain tensor obtained from the macroscale and applied in microscale, $\sigma_{avg}$ is the average stress tensor obtained based on averaging stresses in microscale, ε is the strain tensor in microscale and σ is the stress tensor in macroscale.

In many cases especially when the linear material properties in macroscale are used, the homogenization method can be used for upscaling—the material properties of macroscale material are computed based on prescribed unitary strains in microscale. The micromodel is computed for six cases (for 3D problems) and the average stresses and strains are used for macromodel stiffness tensor evaluation. The constitutive relation between average strain and stress and stiffness tensor is expressed as follows:

(2) $$\sigma_{avg}=C_{h}\varepsilon_{avg}$$

where $C_{h}$ is the stiffness tensor of equivalent homogeneous material that satisfies the elastic deformation characteristic of the heterogeneous material.

In the homogenization problems, three types of boundary conditions can be used: periodic, Dirichlet (displacement), or Neumann (traction). When the size of RVE is sufficiently large, the homogenization convergence allows to eliminate geometrical periodicity. Therefore, the Dirichlet boundary conditions are equivalent to the Neumann boundary conditions in the sense of average material properties obtained during homogenization (both approached converge to the same solution) [53]. In the presented case, Dirichlet boundary conditions were applied.

It should be also noted that for particle-based composites with randomly distributed inclusions, the lack of periodicity makes it difficult to identify RVE therefore, Statistical Volume Elements are processed to homogenization [54]. Pingaro et al. [55] improved that process by developing fast statistical homogenization procedure.

**Figure 3:**
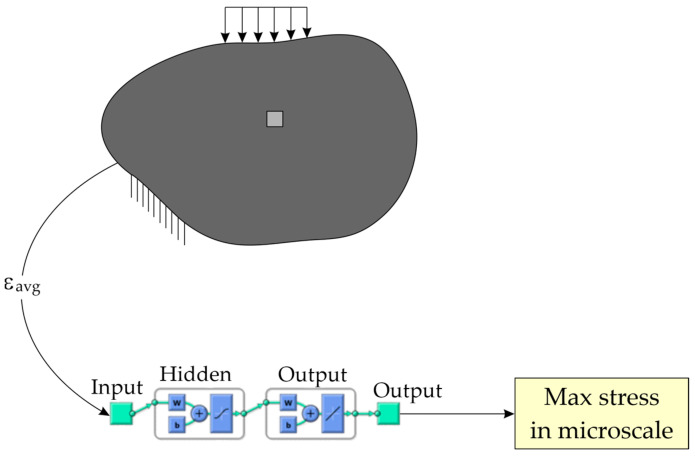
The macro-FEM model and the micro-ANN model.

The localization analysis of micromodel based on strains computed on macroscale is important also in linear problems and allows to computing the maximal stresses in microscale-composite material. The analysis of the micromodel for each average strain tensor can be computationally expensive. The cost of microscale analysis is important, especially in the case of real-time systems monitoring maximum stress levels in structures. As FEM is a computationally demanding method [56,57], to significantly reduce the time needed for microscale model analysis the ANN can be used as a metamodel (Figure 3) replacing the FEM microscale analyses. ANNs can serve as efficient fast surrogate models for problems that require excessive computations. The idea is to use a metamodel instead of the FEM model. The inputs of the metamodel are average strains from the macroscale, while the output is the maximum value of stresses in the micromodel.

ANNs mimic the principles of operation of animal brains and consist of interconnected nodes, or artificial neurons, organized into layers. The signals are transferred from the input layer, through one or more hidden layers, to the output layer. Each node in the input layer represents a feature of the input data. The connections between nodes in adjacent layers have an associated weight. These weights determine the strength of the connection. Additionally, each node in the hidden and output layers has an associated bias. Each node performs a weighted sum of its inputs, adds a bias, and applies an activation function that can be non-linear. The output information of the neurons is passed through the layers until the output is obtained. The network can be trained and learn complex patterns and relationships from provided reference data, by adjusting the weight and bias values of all its neurons [58,59,60].

In the investigated research, the input layer received six average strain components from macroscale and the output layer returned the maximal value of the stress on the microscale. The ANN will work for stress estimation in the considered composite material, at any point, and for any macromodel geometry. The feedforward ANN of the presented architecture works in elastic stress-strain ranges, as elastic-plastic problems are path-dependent [61].

To prepare a reference dataset to train ANNs, a Latin hypercube sampling plan (LHC) was used [62]. The sampling plan was created on six dimensions. The parameter ranges were defined over appropriate and realistic ranges for each selected parameter based on the physical limits of the system and domain knowledge. The number of samples for the LHC plan was determined, considering that the sample size should be large enough to provide a representative data set but should also be manageable within the computational resources. Additionally, randomness was incorporated into the LHC sampling process to avoid predictable patterns in sample selection, which could bias the results.

The microscale boundary value problem was solved for many strain tensors (input vectors for ANN) based on LHC sampling and maximum stress values in microstructure (ANN output) were collected.

To obtain as much accuracy as possible ANN with minimal size, an excessive grid-search hyperparameter optimization was implemented, considering different activation functions and different numbers of hidden neurons.

## 3. Numerical Results

**Figure 4:**
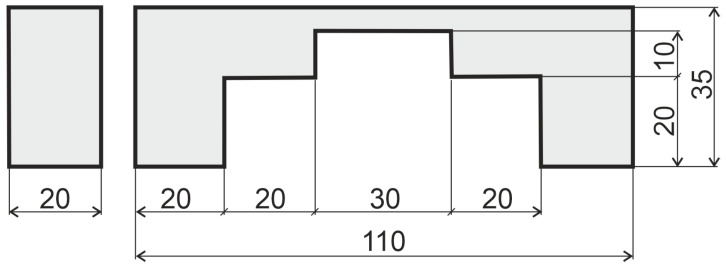
The macroscale geometry of the considered structure.

**Figure 5:**
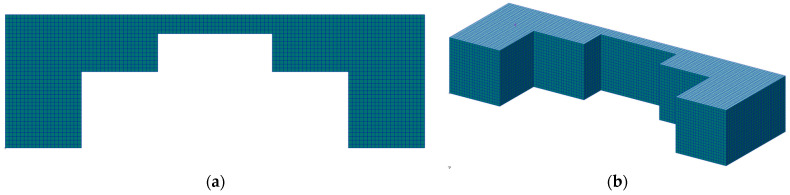
Mesh of the macroscale model: (**a**) front view, and (**b**) isometric view.

**Figure 6:**
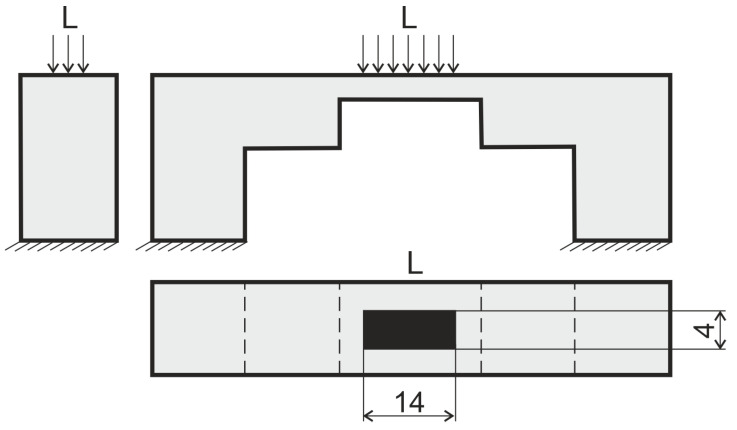
The boundary conditions applied to the macromodel.

Multiscale analysis of a structure made from a glass-epoxy composite is considered as the numerical example. The geometry of the structure in macro scale is shown in Figure 4 and its hexahedral mesh (with about 40,000 elements of size 1 × 1 × 1) is presented in Figure 5. The boundary conditions are shown in Figure 6: two bottom areas are fixed and force is uniformly applied on the marked surface on top of the structure.

**Figure 7:**
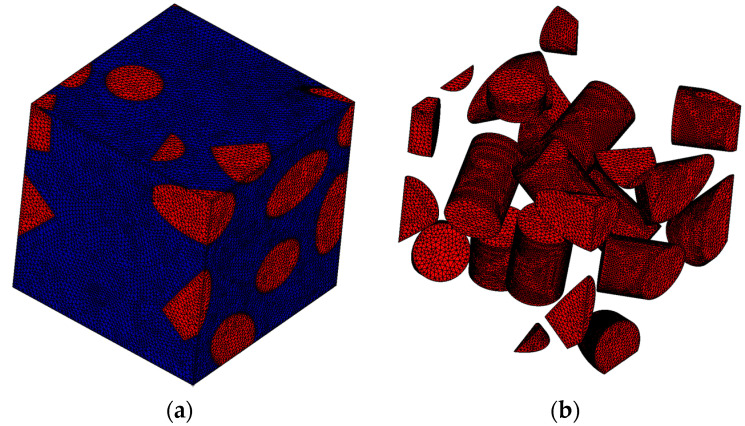
(**a**) The microscale model finite element mesh and (**b**) the fibers.

The micromodel RVE is built from an epoxy matrix and short fiber glass inclusions and is shown in Figure 7. The geometry was created using Material Designer module in ANSYS Workbench 2022R2 software, where the fiber volume fraction was assumed as 0.3. The model was meshed in MSC.Patran 2022.4 software using about 470,000 tetrahedral elements. The material properties are given in Table 1. The MSC.Patran file of the RVE micromodel is available as Supplementary Materials File S1.

In the applied numerical procedure, first the homogenized material properties were determined by RVE analysis in microscale and are presented in Table 2. The RVE was computed for unitary strains in each direction to obtain the average stiffness tensor. Then the homogenized material properties were used in the macroscale. The macroscale model for defined boundary conditions was analyzed and the strain tensors for chosen points were used in microscale analyses to determine the maximum stress values in the composite material. This procedure was performed in two ways: (a) using FEM in the microscale and (b) using previously prepared ANNs as a microscale metamodel.

### 3.1. Results of an Example Multiscale FEM Analysis

**Figure 8:**
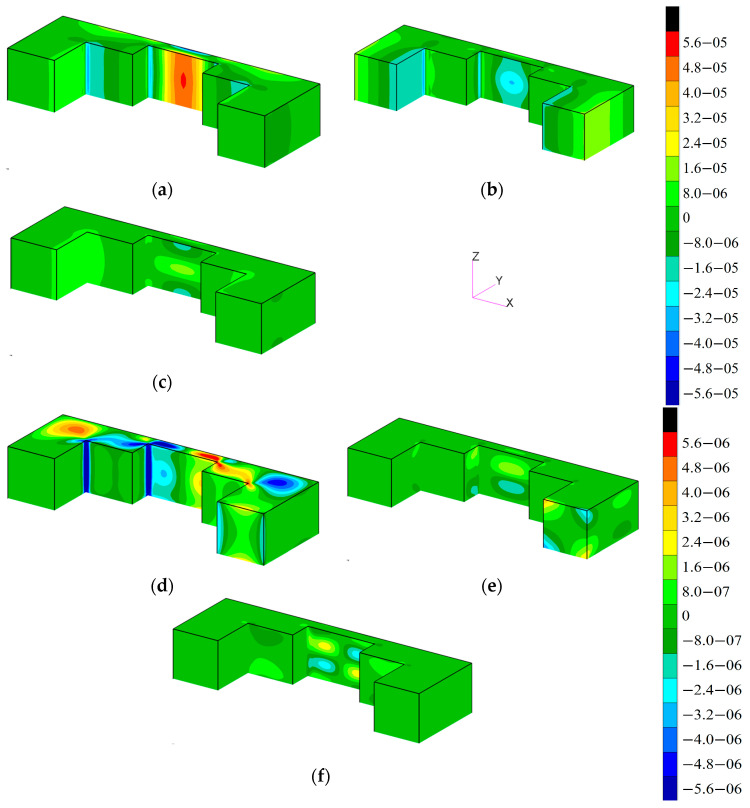
Color map of strains components in the macromodel: (**a**) x, (**b**) y, (**c**) z, (**d**) xy, (**e**) yz, and (**f**) zx.

**Figure 9:**
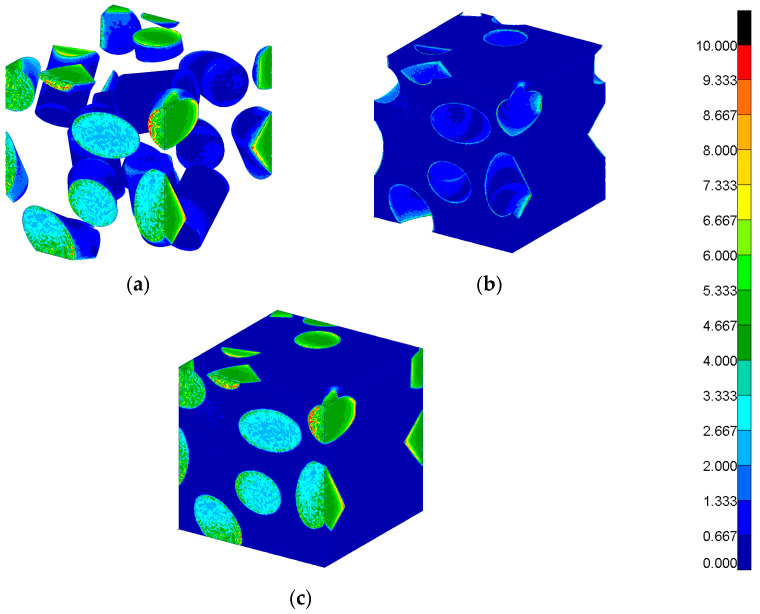
Maps of von Mises stress in the microstructure [MPa]: (**a**) in glass fiber inclusion, (**b**) in the epoxy matrix, and (**c**) in the whole RVE.

In the first case, results are obtained using FEM in both scales. The macromodel was solved, for example, load value and the map of strains in all directions are shown in Figure 8. The strain tensor for the most dangerous areas of the structure was later transferred to the micromodel to obtain local stresses, as shown in Figure 9. The Hexagon/MSC.Nastran 2022.4 solver was used for finite element computations in both scales. Procedures developed by the authors were used for applying boundary conditions of the micromodel by changing the solver input file with values read from relevant results of the macromodel.

### 3.2. Finding the Microscale Metamodel

The metamodels were created to reduce computational cost of microlevel analysis. Two artificial neural networks were trained from the data obtained based on micromodel analyses, according to LHC plan. Each network has six inputs that are independent elements of the strain tensor of microstructure, and one output that is maximal von Mises stress in the inclusions and maximal von Mises stress in the matrix, for the first and second ANN, respectively. To obtain reference data, 4988 microscale finite element analyses were performed, and it took about 4 weeks on 2 processors AMD (Sunnyvale, CA, USA) EPYC 64 core server.

Grid search hyperparameter optimization was performed to obtain the best-fitted ANNs for the given problem. It was assumed that both networks would have one hidden layer and a linear activation function in the output layer. Fifteen different activation functions were considered for the hidden layer: competitive, Elliot symmetric sigmoid, hard limit, symmetric hard limit, log-sigmoid, inverse, positive linear, linear, radial basis, normalized radial basis, saturating linear, symmetric saturating linear, SoftMax, hyperbolic tangent sigmoid, and triangular basis function. Twenty-six different sizes of the hidden layer were considered, from 5 to 30 neurons. For each combination of activation function and hidden layer size (390 combinations), 100 ANN training trials were performed, from which the one that gave the smallest mean squared error (MSE) was for validation data that was not seen during the training. Therefore, for each of the two ANNs, the best of 39,000 trials was chosen. For each trial, 70% of data were used for training, 15% for validation, and 15% for testing. The Levenberg-Marquardt algorithm [63], with random initial solution (normalized with the method of Nguyen and Widrow [64]), was used to train the ANNs.

**Figure 10:**
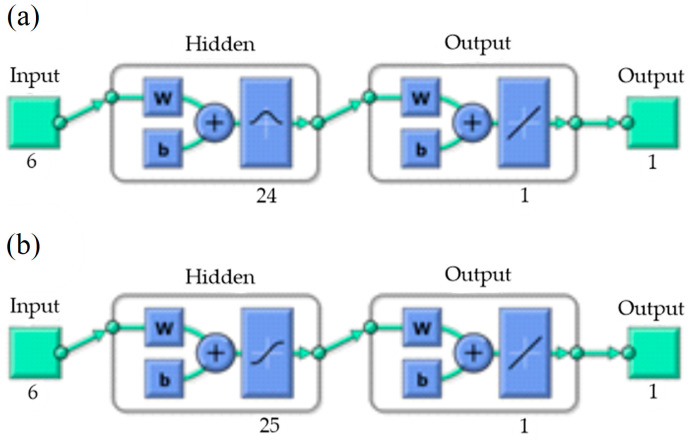
Architectures of the optimized neural networks: (**a**) first ANN, estimating stress in the inclusions and (**b**) second ANN, estimating stress in the matrix.

Architectures of the optimized ANNs are presented in Figure 10. For the first ANN, estimating the maximal von Mises stress in the inclusions, the best parameters were 24 neurons of radial basis activation function in the hidden layer. For the second ANN, estimating the maximal von Mises stress in the matrix, the best parameters were 25 neurons of hyperbolic tangent sigmoid activation function in the hidden layer.

**Figure 11:**
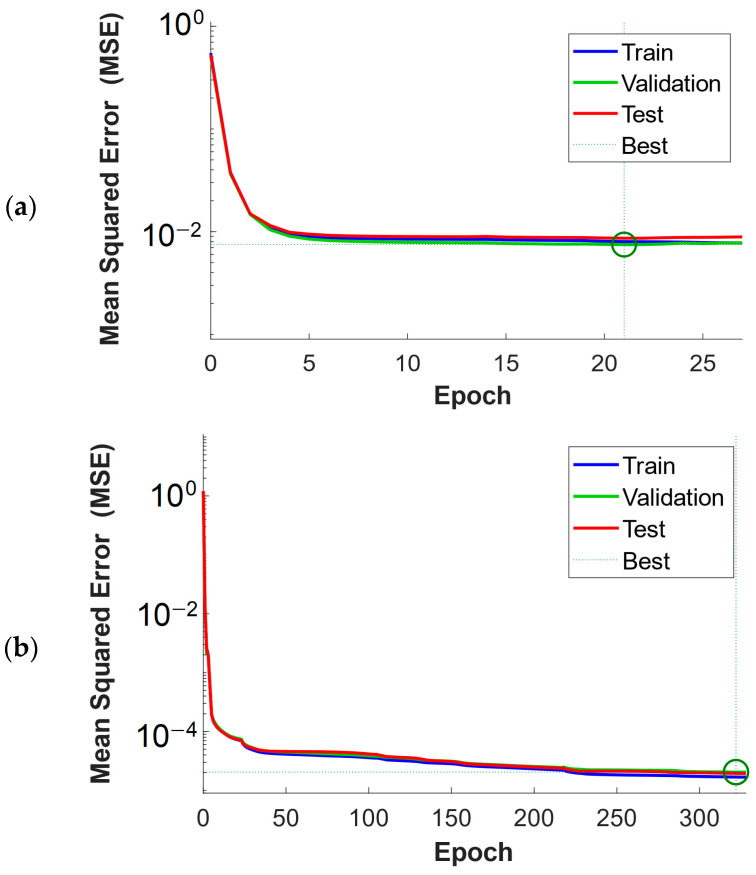
Plots of training performance of the ANNs: (**a**) first ANN, estimating stress in the inclusions and (**b**) second ANN, estimating stress in the matrix.

The plots of the error values of the training record against the number of training epochs, for both networks, are presented in Figure 11. For the first network, the best validation performance of 7.49 × 10^−3^ was achieved at epoch 21. For the second network, the best validation performance of 2.04 × 10^−5^ was achieved at epoch 322. The points of best validation performance were marked with green circles.

### 3.3. Multiscale Analysis with Metamodel

The metamodel created in Section 3.2 was used to determine maximum stress values in the micromodel for different values of loads and points from the macroscale. The accuracy of ANN prediction was tested against exact values from microscale FEM simulations. The results for chosen loads in the macroscale are given in Table 3. The microstresses are calculated for the point with the highest stress value in the macroscale (for homogenized material).

## 4. Discussion

This study focused on advancing the prediction of stress concentrations in composite structures through the application of artificial neural networks to multiscale analysis. The multiscale FEM analyses show that there is a notable reduction in maximum von Mises stress when transitioning from the microscale analysis (which considers the material at a finer level of detail) to the macroscale analysis (which represents a more generalized view of the material). For example, for the first load case presented in Table 3, a difference of one order of magnitude was observed. This highlights the importance of considering multiple scales when evaluating the mechanical behavior of composite materials.

Introducing machine learning (in this case ANNs) as a metamodeling tool for estimating maximal von Mises stress provided a faster and computationally more efficient alternative to the detailed multiscale simulations. Solving the full finite element micromodel on a single processor core took about 26 min while estimating the stresses on a microscale using the presented ANNs can be measured in milliseconds.

Plots of training performance proved that the pattern between strains on the macroscale and stresses on the microscale could be efficiently learned by the utilized ANNs. Grid-search hyperparameter optimization of the ANN architecture allowed us to find as accurate a metamodel as possible. Hyperparameter optimization turned out to be a reasonable approach in the presented case, as the most accurate ANNs did not reach the upper limit of the considered neurons in the hidden layer.

The developed methodology and obtained results consist of a starting point for future research on introducing a multiscale approach combined with machine learning to Structural Health Monitoring and Operational Load Monitoring processes of modern composite structures. The authors believe that implementing metamodels that efficiently describe the microscale behavior of composite materials, based on measurable macroscale features of structures under load, can increase the accuracy of the monitoring processes, especially when microscopic failure mechanisms of composites (microcracks, delamination, etc.) are considered.

Another direction for future research is building a prediction model, based on machine learning, that takes elastic-plastic strains into account. In such cases, the simulation results are loading-path dependent therefore, preparing a training set will be a difficult and time-consuming task. Recurrent networks, including deep network models, will be considered.

## Figures and Tables

**Table 1:** Material properties of composite components.

	Epoxy Matrix	Glass Fibers
Young’s modulus, MPa	3780	78,000
Poison’s ratio	0.35	0.22

**Table 2:** Homogenized material properties for macroscale.

Young’s moduli	E_1_ = 7962.6 MPa	E_2_ = 7951.3 MPa	E_3_ = 7912.7 MPa
Poison’s ratios	ν_12_ = 0.2891	ν_23_ = 0.2874	ν_31_ = 0.2879
Shear moduli	G_12_ = 5863.2 MPa	G_23_ = 5852.6 MPa	G_31_ = 5863.2 MPa

**Table 3:** Maximum von Mises stress values in microscale.

Load Case L [N]	[50, −100, 50]	[100, 0, 0]
$\varepsilon_{x}\;$[$\frac{\mu m}{m}$]	466.1	−120.1
$\varepsilon_{y}\;$[$\frac{\mu m}{m}$]	−265.8	36.4
$\varepsilon_{z}\;$[$\frac{\mu m}{m}$]	161.5	27.5
$\varepsilon_{xy}\;$[$\frac{\mu m}{m}$]	9.31	10.44
$\varepsilon_{yz}\;$[$\frac{\mu m}{m}$]	−19.41	0.04
$\varepsilon_{zx}\;$[$\frac{\mu m}{m}$]	−85.39	3.70
FEM: Max. macro stress [MPa]	6.70	2.96
FEM: Max. micro stress epoxy [MPa]	43.07	8.59
ANN: Max. micro stress epoxy [MPa]	43.02	8.87
FEM: Max. micro stress glass [MPa]	33.30	8.31
ANN: Max. micro stress glass [MPa]	33.11	8.16

## Data Availability

Data is contained within the article and supplementary material.

## Supplementary Materials

The following is available online at https://www.mdpi.com/article/10.3390/ma17010154/s1, File S1: FEM database of the micromodel RVE, to be opened in MSC.Patran software.
